# Development of free 25-hydroxyvitamin D_3_ assay method using liquid chromatography-tandem mass spectrometry

**DOI:** 10.1042/BSR20221326

**Published:** 2022-10-07

**Authors:** Nau Ishimine, Shixing Wu, Ryusei Ota, Koji Takahashi, Masaki Takiwaki, Mitsutoshi Sugano, Minoru Tozuka, Takeshi Uehara

**Affiliations:** 1 Department of Laboratory Medicine, Shinshu University Hospital, Matsumoto, Japan; 2 Division of Medical Technology, School of Health Sciences, Shinshu University School of Medicine, Matsumoto, Japan; 3 Medical Equipment Business Operations, JEOL Ltd., Tokyo, Japan; 4 Department of Clinical Laboratory Sciences, School of Health Sciences, Fukushima Medical University, Fukushima, Japan; 5 Life Science Research Center, Nagano Children’s Hospital, Azumino, Japan; 6 Department of Laboratory Medicine, Shinshu University School of Medicine, Matsumoto, Japan

**Keywords:** free 25(OH)D3, liquid chromatography-tandem mass spectrometry (LC-MS/MS), ultrafiltration, vitamin D metabolites, vitamin D-binding protein (DBP)

## Abstract

The free hormone hypothesis has triggered controversies regarding the measurement of free vitamin D metabolites, such as free 25-hydroxyvitamin D (25(OH)D), as a suitable indicator for total vitamin D for clinical use. This issue can be addressed by developing a precise and accurate method for free 25(OH)D measurement. In the present study, a novel assay method for free 25(OH)D_3_ based on liquid chromatography-tandem mass spectrometry (LC-MS/MS) was developed. Sample preparation first involved ultrafiltration to remove vitamin D-binding protein-bound and albumin-bound 25(OH)D, followed by extraction with a column, derivatization, evaporation, dissolution, and injection into the LC-MS/MS system. The coefficient of variation of repeatability and reproducibility obtained were 3.8–4.5% and 4.8–5.9%, respectively. Satisfactory linearity (*r*=0.999) was obtained up to 80 pg/ml. The lower quantification limit was 0.97 pg/ml and the S/N ratio on the peak of 1.0 pg/ml sample was 24.8 (which is more than the acceptable value of 10). The recovery rate was between 84.5 and 92.4% with a negligible matrix effect (94.5–104.9%). Levels of free 25(OH)D_3_, but not total 25(OH)D_3_, in the serum of the patients with chronic kidney disease (CKD) and hepatic cirrhosis (HC) were substantially lower than those in healthy subjects. The correlation coefficient between total and free 25(OH)D_3_ was 0.738 in all samples, while the linear regression equations were different between the patients with CKD and HC. In conclusion, LC-MS/MS assay for free 25(OH)D_3_ might be useful to evaluate high-throughput methods, including ELISA.

## Introduction

Serum levels of 25-hydroxyvitamin D (25(OH)D) have been widely measured as a biomarker for not only vitamin D deficiency and bone health [1] but also autoimmune diseases [2,3], cardiovascular diseases [3], and cancer [3,4]. Vitamin D_3_, the natural form of vitamin D, is produced from 7-dehydrocholesterol (7-DHC) in the skin by ultraviolet irradiation [5]. Only a small amount of vitamin D is obtained from the diet, with fish containing vitamin D_3_ and plants containing vitamin D_2_. Both vitamin D_3_ and D_2_ (vitamin D) are metabolized by a similar pathway. Vitamin D is hydroxylated in the liver to produce 25(OH)D (25(OH)D_3_ and 25(OH)D_2_), which is the major circulating form and is further hydroxylated by 1α-hydroxylase in the kidney to produce 1α,25-dihydroxyvitamin D (1α,25(OH)_2_D), which is the hormonally active dominant form of vitamin D. The secretion of 1α-hydroxylase is strictly regulated by the three hormones, including stimulation by parathyroid hormone (PTH) and inhibition by fibroblast growth factor 23 (FGF23) and 1α,25(OH)_2_D itself. In circulation, vitamin D and its metabolites are primarily bound to vitamin D-binding protein (DBP) and albumin (Alb), albeit the binding affinity varies depending on the metabolites. 25(OH)D, which has the highest affinity to DBP, exists in three forms with an approximate ratio of 85–90% as DBP-bound, 10–15% as Alb-bound, and 0.03% or less in the free form [6–8].

DBP, synthesized in the liver, strongly binds to 25(OH)D. There are many single-nucleotide polymorphisms (SNPs) in the *DBP* gene [9]. Two dominant SNPs (*rs7041* and *rs4588*) induce three polymorphic alleles and six major phenotypes. These phenotypes of DBP affect the levels of DBP as well as the total 25(OH)D and induce varying binding affinity of each vitamin D metabolite, involving the ratio of free 25(OH)D. DBP is a multifunctional protein and binds to actin released from severely damaged cells to remove it from circulation.

To quantitate 25(OH)D, most clinical laboratories perform fully automated assays based on immunochemical technology. However, the present immunoassays measure total 25(OH)D levels and cross-react with other vitamin D metabolites such as 24,25-dihydroxyvitamin D_3_ (24,25(OH)_2_D_3_) [10]. Recently, some methods using liquid chromatography-tandem mass spectrometry (LC-MS/MS) for measuring vitamin D concentrations have been reported [11–14]. All of these methods were carried out similarly: addition of internal standard, extraction, derivatization, and detection. However, each method has a peculiar ingenuity such as the use of dried blood spots as a sample [11], one-step estimate of bioavailable vitamin D and vitamin D metabolite ratio [12], serum sample preparation using protein precipitation [13], and high-throughput method [14]. The advantage of the LC-MS/MS methods for detection of vitamin D is the ability of the procedure to simultaneously measure multiple vitamin D metabolites, such as 25(OH)D_3_, 3-epi-25-hydroxyvitamin D_3_ (3-epi-25(OH)D_3_), 24,25(OH)_2_D_3_, 1α,25(OH)_2_D_3_, and 25(OH)D_2_.

The free hormone hypothesis [15,16] is a proposition that the free fraction of hormones (i.e., unbound to any protein) can access most target cell cytoplasm by diffusion and express biological effects. The free fraction ratio varies greatly depending on the hormone. For example, the ratio of free thyroxine is 0.03%, while that of free cortisol is 4–10% [16]. It is still controversial whether the free hormone hypothesis applies to vitamin D. However, the total 25(OH)D levels did not necessarily correlate with bone mineral density and risk of fractures [17], which could be indirect evidence of applying the free hormone hypothesis.

At present, free 25(OH)D levels have been determined by two methods-direct quantification using ELISA [18] and calculation using a formula involving a total 25(OH)D, DBP, and Alb concentrations [19]. However, there are some concerns regarding the use of these two methods. First, these two methods often dissociate depending on the target population, since the DBP concentration, which is used for the calculation, varies greatly depending on the physiological condition [5]. Second, cross-reactivity of the specific antibody used in the immunological assays cannot be ruled out and can lead to inaccurate results. Even though they are small, some cross-reactivities are described in the ‘Instructions for Use’ of the ELISA kit for free-25(OH)D (DIASource, Louvain-La-Neuve, Belgium).

To address these limitations, in the present study, we developed a novel method based on LC-MS/MS in combination with ultrafiltration of serum samples, to quantitate and determine free 25(OH)D_3_ as a dominant free vitamin D metabolite. We also compared free and total 25(OH)D_3_ levels in the serum of healthy volunteers (NOR) with that of patients with chronic kidney disease (CKD), hepatic cirrhosis (HC), and pregnant (PRG) women, as the measurement of free 25(OH)D_3_ is useful in understanding the pathophysiology of these conditions [20–22].

## Methods

### Serum samples

The residual serum samples of the patients with CKD and HC and of PRG women obtained from the Clinical Laboratory of Shinshu University Hospital (3-1-1 Asahi, Matsumoto 3908621, Japan) were used to quantitate the total and free 25(OH)D_3_, DBP, and Alb concentrations. Serum samples of NOR were also obtained for LC-MS/MS method development, analytical validation, and for use as the control sample to compare with the test samples. These samples were stored at –80°C until use. The study protocol involving the opt-out consent procedure was approved by the ethics committees of the Faculty of Medicine, Shinshu University (No. 4928).

### Reagents

JeoQuant™ (JEOL Ltd., Tokyo, Japan) was used to quantitate total and free 25(OH)D_3_ levels. Distilled water, acetonitrile, formic acid (LC-MS grade), hexane, and ethyl acetate (for solid-phase extraction) were purchased from FUJIFILM Wako Pure Chemical Corporation (Osaka, Japan).

### Sample preparation for measuring total 25(OH)D_3_

Briefly, 50 μl of serum samples and standards were mixed with 250 μl of the internal standard (IS) solution, including 25(OH)D_3_-^13^C_5_, and applied to a column of solid–liquid extraction (SLE) (ISOLUTE^®^ SLE+; Biotage, Uppsala, Sweden). After 5 min of incubation, 25(OH)D_3_ was eluted using 600 μl of ethyl acetate/hexane (50/50 v/v) three times followed by evaporation at 60°C using Centrifugal Evaporator CVE-3110^®^ (TOKYO RIKAKIKAI CO., LTD, Tokyo, Japan). The residue was then derivatized using 14-(4-(dimethylamino)phenyl)-9-phenyl-9,10-dihydro-9,10-[1,2]epitriazoloanthracene-13,15-dione (DAP-PA) ethyl acetate solution as described previously [23]. After 15 min of derivatization and evaporation at 60°C, the residue was dissolved in 25 μl of 50% (v/v) acetonitrile, and 5 μl of aliquot was injected into the LC-MS/MS system.

### Sample and standard preparation for measuring free 25(OH)D_3_

To separate the free 25(OH)D_3_ fraction in serum, 700 μl of serum sample was loaded onto the ultrafiltration device Amicon® Ultra-2 30k Centrifugal Filter Unit (Merck, Darmstadt, Germany), and centrifuged at 2000× ***g*** for 30 min at 7°C. The filtrate obtained was used as the sample. Standard solutions for free 25(OH)D_3_ were prepared by adequately diluting the JeoQuant™ calibrator (stock solution) with 30% v/v acetonitrile (finally corresponding to 1.1, 10.975, 21.95, and 43.9 pg/ml of free 25(OH)D). Then, 300 μl of standard solution and filtrate were mixed with 80 μl of 100-fold diluted IS solution with 30% (v/v) acetonitrile, followed by extraction with SLE column, derivatization using DAP-PA, and evaporation as described above. The residue was dissolved in 20 μl of 50% (v/v) acetonitrile and 5 μl of the aliquot was injected into the LC-MS/MS system.

### LC-MS/MS conditions

The pretreated samples were analyzed using an ekspert™ microLC 200 System coupled to a TripleTOF™ 4600 System (SCIEX, Vaughan, ON, Canada). Chromatographic separation was performed using the CAPCELL CORE C18 column (1.0 mm i.d. ×100 mm) (OSAKA SODA, Osaka, Japan) with binary mobile phases, water (Eluent A) and acetonitrile (Eluent B), both containing 0.1% (v/v) formic acid. The flow rate and total run time were set at 50 μl/min and 8 min, respectively. The gradient conditions were as follows: 0–0.1 min (30% Eluent B), 0.1–0.8 min (linear gradient to 58% Eluent B), 0.8–3.67 min (linear gradient to 71% Eluent B), 3.67–5.17 (95% Eluent B), and 5.17–8 min (30% Eluent B for re-equilibration of the column). The column and autosampler temperatures were maintained at 40°C and 10°C, respectively. Samples were ionized using the positive electrospray ionization (ESI+) source under a capillary voltage of 5500 V and desolvation temperature of 400°C. Selected reaction monitoring (SRM) was used for the quantification. The SRM transitions (m/z) were monitored as follows: 619.3→341.1 for derivatized 25(OH)D_3_ and 624.5→341.1 for derivatized 25(OH)D_3_-^13^C_5_.

### Validation of LC-MS/MS assay for free 25(OH)D_3_

Free 25(OH)D_3_ levels were measured for three different concentrations of diluted JeoQuant™ calibrator (Low: 3.0 pg/ml, Middle: 15 pg/ml, High: 30 pg/ml) to evaluate repeatability (*n*=5) and reproducibility (*n*=25, five replicates for 5 days). The linearity was evaluated using four samples prepared with an adequately diluted JeoQuant™ calibrator. The lower limit of quantification (LLoQ) was determined as the lowest concentration with less than 20% coefficient of variation (CV) according to the repeated measurement (*n*=10, duplicate for 5 days) of five samples prepared with a serially diluting JeoQuant™ calibrator. Matrix effects were assessed by spiking 80 μl of two different concentrations of IS (100 and 500 pg/ml of 25(OH)D_3_-^13^C_5_) into 1.2 ml of ethyl acetate/hexane (1:1, v/v) and 1.2 ml of the SLE column eluent obtained by the serum sample preparation process for 25(OH)D_3_ measurement. The matrix effect was calculated as the percentage of peak area for IS added to the filtrate obtained by ultrafiltration of pooled serum to that added to ethyl acetate/hexane (1:1, v/v). The recovery rate of the SLE column was evaluated by adding 20 μl of standard solutions (0, 110, and 220 pg/ml) to 300 μl of the filtrate obtained by ultrafiltration of pooled serum.

### ELISA for measurement of free 25(OH)D and DBP levels

Serum concentrations of free 25(OH)D and DBP were measured using commercially available ELISA kits (DIAsource ImmunoAssays; and Immundiagnostik AG, Bensheim, Germany, respectively) by following each manufacturer’s protocol. The CV of repeatability (intra-assay) and reproducibility (interassay), described by the manufacturers, are 1.9–5.5% and 4.0–6.3 for free 25(OH)D and 3.3–5.0%, 3.3–13.9% for DBP, respectively.

### Measurement of Alb levels

Alb concentration in serum was determined using the bromocresol purple (BCP) method with a commercially available kit (CicaLiquid ALB-P, Kanto Chemical Co., Inc., Tokyo, Japan).

### Calculation of free 25(OH)D levels

Free 25(OH)D concentration was calculated based on the determined levels of total 25(OH)D_3_, DBP, and Alb using the formula described by Bikle et al. [free 25(OH)D = total 25(OH)D/(1+(6 × 10^5^ × albumin) + (7 × 10^8^ × DBP))] [19].

### Data and statistical analyses

LC-MS/MS data were analyzed using Multi Quant software (SCIEX). The correlation between free and total 25(OH)D_3_ concentrations was evaluated using the Pearson correlation coefficient. Mann–Whitney U-test was used to evaluate the statistical significance of free and total 25(OH)D_3_ concentrations in patients with CKD or HC and PRG women compared with those in NOR. A *P*-value < 0.05 was considered statistically significant.

## Results

### Validation of LC-MS/MS assay for measuring free 25(OH)D_3_ concentration

**Figure 1:**
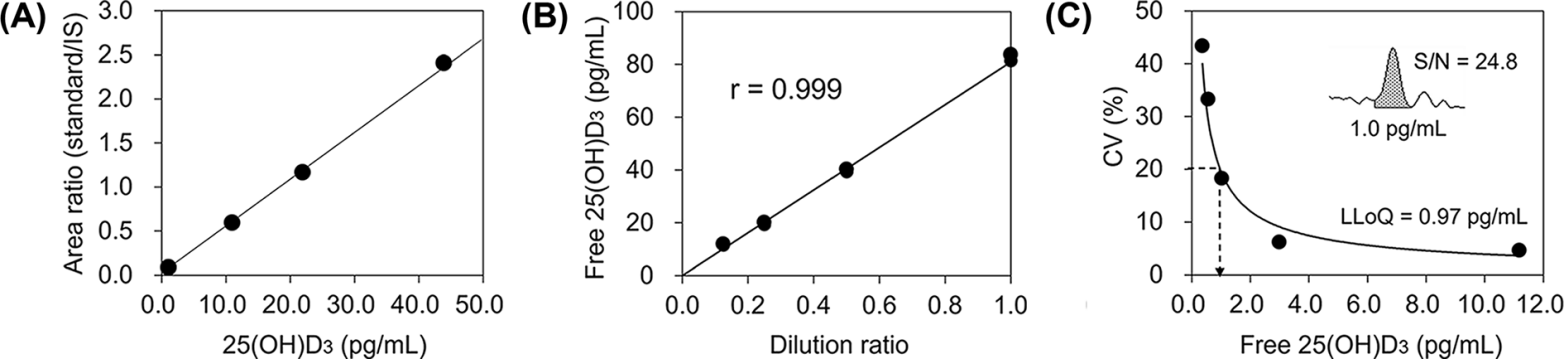
Validation of LC-MS/MS assay for measuring free 25(OH)D_3_ concentration (**A**) Calibration curves were generated using four standards corresponding to 1.1, 10.975, 21.95, and 43.9 pg/ml of free 25(OH)D_3_ based on the ratio of the peak area of each standard to that of the corresponding IS. (**B**) The linearity was evaluated according to the CLSI guideline using four samples prepared by adequately diluting the JeoQuant™ calibrator. (**C**) The LLoQ was determined as the lowest concentration with less than 20% CV according to the repeated measurement (*n*=10, duplicate for 5 days) of five samples prepared by serially diluting the JeoQuant™ calibrator. The inside figure indicates the peak obtained by LC-MS/MS for 1 pg/ml of free 25(OH)D_3_ (S/N ratio: 24.8).

Calibration was performed using four standards corresponding to 1.1, 10.975, 21.95, and 43.9 pg/ml of free 25(OH)D_3_. A calibration curve was generated based on the ratio of the peak area of each standard to that of the corresponding IS (Figure 1A). The ratio of the peak area was proportional to the concentration of free 25(OH)D_3_. The CV (%) of repeatability and reproducibility of the three samples were 3.8–4.5% and 4.8–5.9%, respectively (Table 1). Satisfactory linearity (*r*=0.999) was observed at least up to 80 pg/ml (Figure 1B). LLoQ with a CV of 20% was 0.97 pg/ml and the S/N ratio (24.8) on the peak of 1.0 pg/ml sample was more than the acceptable value of 10 (Figure 1C). The recovery rate after subjecting the filtrate of ultrafiltration to the SLE column was between 84.9% and 92.4% and the matrix effect was negligible (94.5–104.9%).

**Table 1:** Repeatability and reproducibility of LC-MS/MS assay for free 25(OH)D3

	Mean ± SD (pg/ml)	CV (%)	Mean ± SD (pg/ml)	CV (%)
Low	3.02 ± 0.14	4.5	2.99 ± 0.14	4.8
Middle	15.54 ± 0.61	3.9	14.96 ± 0.85	5.7
High	31.67 ± 1.20	3.8	30.09 ± 1.77	5.9

* Five replicates for 5 days.

### Total and free 25(OH)D_3_ levels in patients with CKD and HC and PRG women

**Figure 2:**
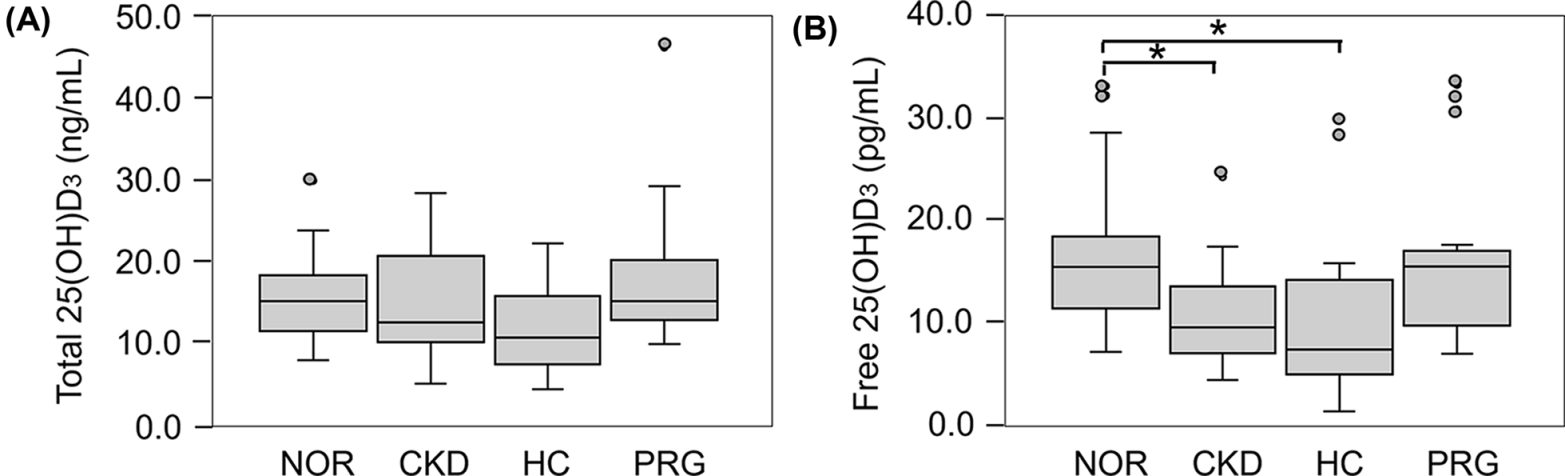
Distribution of total and free 25(OH)D_3_ levels in the patients with CKD and HC and PRG women Box plots indicate the distribution of (**A**) total and (**B**) free 25(OH)D_3_ levels in serum obtained from the patients with CKD (*n*=15) and HC (*n*=15), as well as PRG (*n*=15). Mann–Whitney U-test was used for data analysis between NOR (*n*=15) and test individuals; *P<0.05.

**Figure 3:**
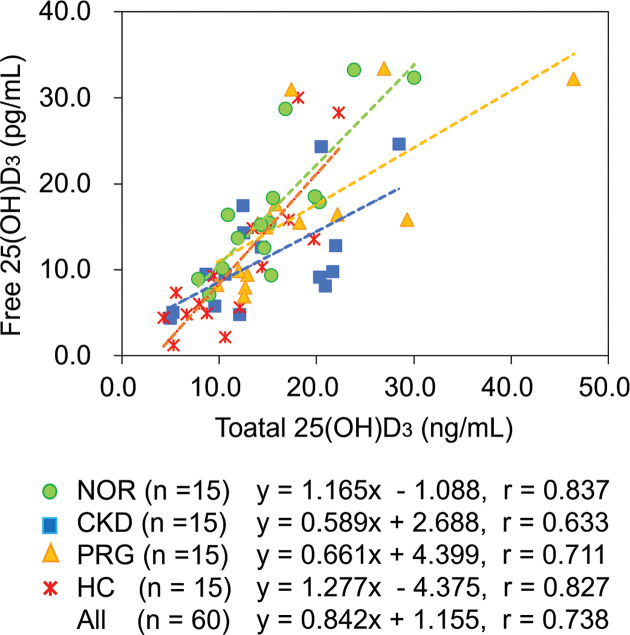
Correlation between total and free 25(OH)D_3_ levels The correlation between total and free 25(OH)D_3_ levels were evaluated for subjects from all groups (NOR, CKD, HC, and PRG). The linear regression equation and the correlation of coefficient of each group are indicated under the correlation diagram.

The distribution of total and free 25(OH)D_3_ levels in serum obtained from patients (*n*=15 in each group) with CKD, HC, and PRG women was compared with those obtained from the NOR group (*n*=15). No remarkable difference in total 25(OH)D_3_ levels were observed between NOR and any test groups (Figure 2A). In contrast, free 25(OH)D_3_ levels in the patients with CKD (median: 9.51 pg/ml, IQR: 6.36–13.93 pg/ml) and HC (median: 7.32 pg/ml, IQR: 4.85–14.54 pg/ml) were substantially lower than those of the NOR group (median: 15.47 pg/ml, IQR: 10.78–18.51 pg/ml) (Figure 2B). Relatively strong correlation (*r*=0.738) between free (y) and total (x) 25(OH)D_3_ levels for all subjects was observed with linear regression equation of *y*=0.842x + 1.155 (Figure 3). However, the linear regression equation for each disease/condition was different from that of NOR; particularly, the slopes of CKD (0.589) and PRG (0.661) were less than those of NOR (1.165).

### Correlation between calculated free 25(OH)D and total and free 25(OH)D_3_ levels measured using LC-MS/MS

**Figure 4:**
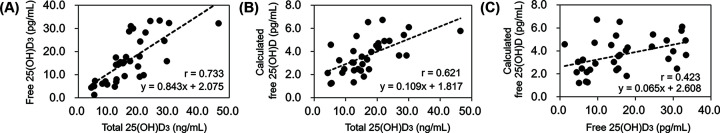
Correlation between calculated free 25(OH)D and LC-MS/MS-measured total and free 25(OH)D_3_ levels Free 25(OH)D levels calculated using the formula described by Bikle et al. [19] were compared with free and total 25(OH)D_3_ measured by LC-MS/MS for a part of subjects (*n*=34) whose DBP and Alb levels in serum were known. Correlations were evaluated between (**A**) free and total 25(OH)D_3_ levels, (**B**) calculated 25(OH)D and total 25(OH)D_3_ levels, and (**C**) calculated 25(OH)D and 25(OH)D_3_ levels.

Total and free 25(OH)D_3_ levels in serum samples obtained from subjects (*n*=34), for whom data regarding DBP and Alb levels were available, were compared with the calculated free 25(OH)D levels (Figure 4). It was confirmed that the correlation coefficient between total and free 25(OH)D_3_ levels (*r*=0.733) and linear regression equation (*y*=0.843x + 2.075) were approximate to those obtained from all subjects (*n*=60, *r*=0.738, *y*=0.842x + 1.155) indicated in Figure 3 (Figure 4A). The correlation coefficient between calculated free and total 25(OH)D levels (Figure 4B) or measured free 25(OH)D_3_ levels (Figure 4C) were 0.621 and 0.423, respectively.

### Comparison between the absolute values of 25(OH)D determined using LC-MS/MS and ELISA

To evaluate the relevance of the absolute values of free 25(OH)D_3_ determined using the LC-MS/MS and ELISA methods [for 25(OH)D], the filtrate of ultrafiltration obtained from pooled serum, with 20.96 ng/ml of total 25(OH)D_3_ level, was measured using both methods (Table 2). A large difference was observed between the results of the two methods (16.71 pg/ml using LC-MS/MS and 0.46 pg/ml using ELISA). Adequately diluted standards used in LC-MS/MS method (10 and 20 pg/ml) were also measured in ELISA and extremely small values (0.39 and 0.43 pg/ml, respectively) were obtained. Different amounts of the top fractions (0, 20, 40, and 60 μl) after ultrafiltration (in other words concentrated serum), in which the total 25(OH)D_3_ was 44.54 ng/ml, were added to 240 μl of filtrate after ultrafiltration and adjusted to 300 μl of final volume by adding saline. Free 25(OH)D concentration in each sample measured using ELISA did not indicate a linear regression (0.46, 2.61, 3.29, and 3.74, respectively) (Table 2). The total 25(OH)D_3_ concentration in a mixture of 240 μl of filtrate and 60 μl of the top fraction was 9.44 ng/ml.

**Table 2:** Comparison between LC-MS/MS assay and ELISA for free 25(OH)D_3_

	LC-MS/MS		ELISA
	Total 25(OH)D_3_ (ng/ml)	Free 25(OH)D_3_ (pg/ml)	Free 25(OH)D (pg/ml)
Pooled serum	20.96	-	3.27
Concentrated serum^*^	44.54	-	-
Filtrate + concentrated serum + saline			
240 + 0 + 60 (μl)	-	16.71	0.46
240 + 20 + 40 (μl)	-	-	2.61
240 + 40 + 20 (μl)	-	-	3.29
240 + 60 + 0 (μl)	9.44	-	3.74
LC-MS/MS standard (10 pg/ml)	-	-	0.39
LC-MS/MS standard (20 pg/ml)	-	-	0.43

* Top fraction after ultrafiltration.

## Discussion

The free hormone hypothesis [15,16] has triggered controversies regarding whether assaying free vitamin D metabolites, such as free 25(OH)D, is more relevant for clinical use compared with estimating total vitamin D levels. Recently, an ELISA system for measuring free 25(OH)D with a relatively high-throughput screening ability and adequate reproducibility was developed. Although ELISA can facilitate the progress of research related to free 25(OH)D, the accuracy of the method (in terms of absolute values) needs to be further clarified for its global application in clinical laboratories. One way to improve the accuracy would be to develop novel measurement methods using other principles. The LC-MS/MS method is considered to be a sensitive, highly specific, and accurate method; however, it is essential to separate the free 25(OH)D fraction from the total 25(OH)D fraction, which includes the DBP- and Alb-bound 25(OH)D. Equilibrium dialysis is known to be the most adequate method to separate the free fraction, although it is time-consuming. We developed a novel method in which ultrafiltration was used to separate free vitamin D metabolites, followed by a measurement of free 25(OH)D_3_ as a dominant free vitamin D metabolite using the LC-MS/MS system.

The basic performance of the LC-MS/MS method, such as repeatability, reproducibility, linearity, and LLoQ, was satisfactory, along with the sample preparation steps. The lower limit of detection (LLoD) of the ELISA kit for 25(OH)D is described as 2.4 pg/ml in the ‘Instructions for Use’ (DIASource). LLoQ is generally larger than LLoD, suggesting that the sensitivity of our LC-MS/MS method for free 25(OH)D_3_ measurement is at least more than two times higher than that of the ELISA method. The recovery rate after subjecting the sample to SLE column extraction showed a slightly wider range (84.9–92.4%). However, considering the CV (%) of repeatability and reproducibility (3.8–4.5% and 4.8–5.9%, respectively), the range of recovery rate in SLE column extraction is thought to be acceptable. No matrix effect was observed in LC-MS/MS assay at the final step of this method. In conclusion, the present method was found to be satisfactory for free 25(OH)D_3_ measurement.

The total and free 25(OH)D_3_ levels in serum were measured for 60 subjects included in the NOR (*n*=15), CKD (*n*=15), HC (*n*=15), and PRG (*n*=15) groups since the latter three conditions are thought to be associated with vitamin D levels in plasma [20–22]. The total 25(OH)D_3_ levels (median: 14.30, IQR: 10.47–19.79) were consistent with that of a previous report (median = 11.88 ng/ml, IQR: 7.68–17.68 ng/ml), in which the total 25(OH)D_3_ levels were also measured using the LC-MS/MS [24]. No remarkable difference was observed in a total 25(OH)D_3_ levels between NOR and other test groups. However, the free 25(OH)D_3_ serum levels in CKD and HC groups were substantially lower than that in the NOR group, indicating the possibility that the measurement of free 25(OH)D_3_ is useful to understand the pathophysiology of the condition. The correlation coefficient between total and free 25(OH)D_3_ for all subjects (*n*=60) indicated a relatively high correlation (*r*=0.738), which is consistent with a previously described result (*r*=0.77) obtained using ELISA for free 25(OH)D and chemiluminescent immunoassay (CLIA) for total 25(OH)D measurements [25]. However, the linear regression equation of correlation between total and free 25(OH)D levels was different under each condition. Although the linear regression equation of correlation between total and free 25(OH)D is disease-specific in clinical populations [26], there is a discrepancy in the findings including ours [27–30]. The discrepancy could be attributed to differences in the assay methods used for the measurement of 25(OH)D in the studies. First, as can be seen in the formula used to obtain free 25(OH)D [free 25(OH)D = total 25(OH)D/(1+(6 × 10^5^ × albumin) + (7 × 10^8^ × DBP))], the calculated free 25(OH)D levels could be lower in PRG subjects than in NOR subjects and patients with liver failure. The DBP levels are known to be significantly higher in PRG subjects than in normal subjects [31], even if the albumin levels in PRG subjects are only slightly lower than those in normal subjects [32]. On the other hand, both DBP and albumin levels in liver failure patients are lower than those in normal subjects [27,32]; however, total 25(OH)D levels are also low in patients with liver failure [27,28]. Consequently, no significant difference was observed in calculated free 25(OH)D levels between normal subjects and patients with liver failure. Even though it is a convenient method, these findings imply the inaccuracy of the calculated free 25(OH)D values in subjects with some disorders. This could probably be the reason for the low correlation coefficient (*r*=0.423) observed in free 25(OH)D measured by LC-MS/MS and that calculated by the formula in the present study. A relatively low correlation coefficient was also observed in a previous report (*r*=0.553) [33]. Second, free 25(OH)D levels measured by the ELISA method have been reported to be lower in PRG subjects and higher in patients with liver failure patients when compared with those in the normal subjects [27,28]. In contrast, the measurement of free 25(OH)D_3_ levels using the present LC-MS/MS method revealed completely different results. Free 25(OH)D_3_ levels in normal and PRG subjects were almost similar and relatively higher than those in patients with liver failure. Third, the previously indicated correlation between total 25(OH)D measured by LC-MS/MS (on the *x*-axis) and free 25(OH)D measured by ELISA (on the *y*-axis) indicated that the slopes of linear regression equations of both normal and PRG subjects were similar, whereas a relatively larger slope of linear regression was obtained for patients with liver failure [28]. However, in the present study, the linear regression equations of both normal subjects and patients with liver failure had similar slopes, whereas the linear regression equations of PRG subjects had a comparatively lower slope. We speculate that the sample dilution required for the ELISA method to measure free 25(OH)D might influence the equilibrium between free and bound 25(OH)D depending on the concentration of DBP, leading to the aforementioned difference.

Free 25(OH)D levels in serum are determined using the ELISA as a direct measurement method [25,28,34,35] and calculation formula [6,28]. Free 25(OH)D levels in serum measured using the ELISA were indicated as follows (mean ± SD): 4.73 ± 1.54 pg/ml [25], 4.5 ± 1.6 pg/ml [28], 8.05 ± 4.66 pg/ml (mean ± SD) for subjects with various ages [34], and median 4.32 pg/ml and IQR 3.29–5.72 pg/ml [35]. Meanwhile, the calculated free 25(OH)D levels in serum were as follows: median 22.0 pmol/L (8.8 pg/ml) and IQR 16.5–26.2 pmol/L (6.6–10.5 pg/ml) [6] and 7.7 ± 4.3 pg/ml (mean ± SD) [28]. In contrast, the free 25(OH)D_3_ levels obtained using the LC-MS/MS method in the present study (*n*=60) were 14.0 ± 8.4 pg/ml (mean ± SD) and range 4.4–33.3 pg/ml. To evaluate the reason for this discrepancy, free 25(OH)D_3_ concentration in the filtrate of ultrafiltration obtained from pooled serum was measured using the ELISA. The obtained result (0.46 pg/ml) was substantially lower than that obtained using the LC-MS/MS (16.71 pg/ml). Free 25(OH)D concentration of the pooled serum measured directly using the ELISA was 3.27 pg/ml. Free 25(OH)D_3_ is a dominant fraction of free vitamin D, meaning free 25(OH)D is almost the same as free 25(OH)D_3_ levels. In addition, the standards for LC-MS/MS corresponding to 10 and 20 pg/ml of free 25(OH)D_3_ were estimated as 0.39 and 0.43 pg/ml using the ELISA, respectively. The ratio of dissociation is nearly equal to that obtained by the measurement of filtrate after ultrafiltration. Total 25(OH)D_3_ levels in the top fraction after ultrafiltration (concentrated serum) was 44.54 ng/ml, which is reasonable because the pooled serum with 20.96 ng/ml of total 25(OH)D_3_ was concentrated approximately twofold by ultrafiltration. The fluctuation in the free 25(OH)D_3_ levels obtained by adding different volumes of the top fraction is difficult to interpret. At least, total 25(OH)D_3_ level of the mixture (9.44 ng/ml) containing 60 μl of concentrated serum and 240 μl of filtrate after ultrafiltration is a reasonable result. If the data on free 25(OH)D obtained using the ELISA are accurate, the extremely high values obtained using LC-MS/MS could be induced by ultrafiltration for 30 min. This could be caused by a contamination of 25(OH)D_3_ bound to DBP or Alb, or a loss of balance and equilibrium between the free and bound fractions. However, considering the ELISA results as indicated in Table 2, it is difficult to say whether ELISA is accurate. To measure free 25(OH)D in the presence of more than 1000-fold of 25(OH)D bound to DBP or Alb is not easy using the LC-MS/MS as well as the ELISA method. The possibility cannot be denied that contamination and unexpected reaction including cross-reaction induced by a large amount of 25(OH)D_3_ bound to DBP and Alb may affect the measurement accuracy of the LC-MS/MS and ELISA methods, respectively.

The LC-MS/MS assay for estimating free 25(OH)D_3_ described here is not suitable for use in clinical laboratories because it requires a large amount of serum and is a complicated process. However, the LC-MS/MS assay, which has high sensitivity and selectivity for free 25(OH)D_3_ measurements, might be a useful method to evaluate high-throughput methods, including ELISA. In conclusion, the LC-MS/MS method for measuring free 25(OH)D_3_ levels can aid in future research involving clinical populations.

## Data Availability

All data supporting the findings of the present study are available from the corresponding author (Takeshi Uehara, tuehara@shinshu-u.ac.jp) upon reasonable request.

## Abbreviations

7-DHC: 7-dehydrocholesterol
25(OH)D: 25-hydroxyvitamin D
Alb: albumin
BCP: bromocresol purple
CKD: chronic kidney disease
CLIA: chemiluminescent immunoassay
CLSI: Clinical and Laboratory Standards Institute
CV: coefficient of variation
DAP-PA: 14-(4-(dimethylamino)phenyl)-9-phenyl-9,10-dihydro-9,10-[1,2]epitriazoloanthracene-13,15-dione
DBP: vitamin D-binding protein
ELISA: enzyme-linked immunosorbent assay
ESI: electrospray ionization
FGF23: fibroblast growth factor 23
HC: hepatic cirrhosis
IQR: interquartile range
IS: internal standard
LC–MS/MS: liquid chromatography-tandem mass spectrometry
LLoD: lower limit of detection
LLoQ: lower limit of quantification
NOR: healthy volunteers
PRG: pregnant
PTH: parathyroid hormone
SD: standard deviation
SLE: solid–liquid extraction
SNP: single-nucleotide polymorphisms
SRM: selected reaction monitoring
